# It’s a Question at the ‘Root’ of the Problem: Fungal Associations of *Dionaea muscipula* (Venus’ Flytrap) Roots in Its Native Habitat

**DOI:** 10.3390/microorganisms13102269

**Published:** 2025-09-27

**Authors:** Anna A. Carnaggio, Michelle M. Barthet

**Affiliations:** 1 Department of Biology, Coastal Carolina University, Conway, SC 29526, USA; aacarnagg@coastal.edu; 2 Coastal Marine and Wetland Studies, Coastal Carolina University, Conway, SC 29526, USA

**Keywords:** *Dionaea muscipula*, Venus’ flytrap, carnivorous plants, fungal endophytes, mycorrhizae

## Abstract

Carnivorous plants survive in harsh habitats with limited nutrients and a low pH. Much focus has been placed on carnivorous trap evolution as the primary mechanism to increase nutrient acquisition through insect digestion. Soil microbiome, however, may also play a pertinent role in nutrient acquisition influencing plant vigor and overall success. *Dionaea muscipula*, commonly known as the Venus’ flytrap, is endemic to rims of the Carolina Bays located in southeast North Carolina and northeast South Carolina, where *D. muscipula* survives in nutrient poor soils with a vestigial root system. We utilized a combination of microscopy, plating, and metagenomics, to investigate the presence/absence of fungal partners that may contribute to success and vigor of *D. muscipula* in its native habitat in order to further conservation of this carnivorous plant. Results support that *D. muscipula* forms both mycorrhizal and fungal endophytic associations, most likely to aid nutrient uptake from otherwise nutrient-poor soils, as well as aid in stress defense. Several ectomycorrhizal, endophytic, and saprophytic fungal species were identified from the surrounding rhizosphere of *D. muscipula* roots presenting a first glimpse into fungal communities that may influence *D. muscipula* physiology and compose the microbiome of the Carolina Bays ecosystem.

## 1. Introduction

*Dionaea muscipula*, commonly known as the Venus’ flytrap, is a plant native to the southeast corner of North Carolina and northeast corner of South Carolina [1,2]. *D. muscipula* in its native habitat is found in wetlands on the rims of Carolina Bays adapted to acidic nutrient poor soils [3] and regular burns for removal of vegetation that would otherwise impede light acquisition [1,2,4]. Although no longer considered federally endangered or threatened, *D. muscipula* continues to be protected in both North and South Carolina due to continual risk of habitat loss, fire suppression, and illegal poaching [5,6,7]. One method utilized to manage *D. muscipula* population numbers is transplantation of native plants into new suitable habitat [8]. Transplantation studies demonstrated *D. muscipula* trap size, production, and ability to capture prey were not impacted by placement in a new site [9] suggesting little importance on soil microbiome for *D. muscipula* establishment. Long-term studies of transplanted *D. muscipula* populations, though, revealed that over a period of six years, transplanted *D. muscipula* populations’ survival and population expansion was highest when transplants were placed in areas that *D. muscipula* once inhabited [8]. These findings suggest that the soil and environment where native populations of *D. muscipula* reside are unique in some aspect that influences plant vigor and is hard to mimic even in nearby locations. We postulated that fungal associations such as mycorrhizae and fungal endophytes aid viability and vigor of *D. muscipula* in its native soils. Such associations may have significant impact on conservation practices of native *D. muscipula*.

Many plants form associations with mycorrhizal fungi in order to increase nutrient absorption through the roots by increasing surface area due to the extensive hyphal network associated with these fungal partners (e.g., [10,11]). Examples include oak, juniper [12], tomatoes [12,13], and magnolia [14]. Contrary to the utilization of mycorrhizae for nutrient acquisition, some plants do not host mycorrhizae in root structures but instead rely on increased branch structure and/or root hairs to optimize nutrient absorption from soils for plant development and success. A prime example is the model plant species *Arabidopsis thaliana* (reviewed in [15]). However, even plants known to not utilize mycorrhizae for nutrient uptake may form temporary mycorrhizal associations with very different functions. Mycorrhizae are known to aid in protection from drought [16], pathogen defense (e.g., [17]), and tolerance to acidic soils [18]. Furthermore, many plants only form mycorrhizal associations when exposed to fungal networks of host plant species [19]. For example, arbuscular mycorrhizal fungi (AMF) of the genus *Rhizophagus* were shown to colonize roots of *Arabidopsis*, a known nonhost plant for mycorrhizae, when *Arabidopsis* was grown along with *Medicago*, an AMF host plant, but not when *Arabidopsis* was grown alone [15,19]. The association of *Rhizophagus* to nonhost *Arabidopsis* roots, however, was antagonistic resulting in reduced growth and increased expression of defense genes as compared to AMF *Rhizophagus* interactions with host plant *Medicago* which increased carbon metabolism and nutrient transport [19]. Thus, it is possible that several other presumed AMF nonhost plants form temporary fungal associations when either exposed to host plant fungal networks or when exposed to abiotic or biotic stress, albeit these AMF associations may result in antagonistic, unbeneficial effects.

*D. muscipula* habitat includes acidic soils with low macronutrient content (e.g., nitrogen, calcium, potassium, and phosphorous [3]) resulting in a need to supplement soil nutrient availability by some other mechanism, presumably digestion of prey caught in the evolved leaf traps (reviewed in [20]) and/or fungal associations with root structure to increase surface area for nutrient acquisition. *D. muscipula*, however, has a vestigial root system with a greatly reduced structure relative to non-carnivorous plants [21,22] suggesting decreased ability for roots to function in nutrient uptake from surrounding soils. Contrary to this assumption, Gao et al. [23] determined that the roots of *D. muscipula* were physiologically functional and responsible for the majority of at least one macronutrient, nitrogen, content in the roots. Prey digestion through the evolved leaf trap was found to supply the majority of nitrogen in the foliage, with only a limited amount of nitrogen from prey allocated to roots [23]. Similarly, studies of mineral uptake in the carnivorous sundew species *Drosera capillaris*, *Drosera aliciae*, and *Drosera spathulate* demonstrated that nutrient acquisition through root structure remains the predominant form of obtaining minerals in these carnivorous plants and that the main benefit of carnivory is to stimulate enhanced nutrient uptake from roots [21]. These studies provide strong support for the roots of carnivorous plants such as *D. muscipula* to not only be functional but vital for nutrient acquisition. As such, mycorrhizal associations would be highly beneficial to enable optimum nutrient acquisition for long-term establishment in otherwise nutrient-poor soils. Studies using greenhouse grown carnivorous plants including *D. muscipula* demonstrated mycorrhizal associations, specifically symbiosis with arbuscular mycorrhizal fungi as evident from AMF structures observed using microscopy [24]. Although the presence of AMF in greenhouse grown *D. muscipula* roots suggest that *D. muscipula* is a host plant for mycorrhizal associations, whether these associations occur naturally or were induced due to proximity of other plants in the same greenhouse or other environmental influence of the greenhouse itself remains unclear. No study, to our knowledge, has yet investigated mycorrhizal or other root fungal associations of native *D. muscipula*.

Fungal endophytes may also contribute to the success of *D. muscipula* in its native environment. Fungal endophytes are fungi that form a symbiotic relationship with host plant tissues. Unlike mycorrhizae, however, fungal endophytes are not restricted to the root but instead can be found throughout the host plant depending on the type of endophyte. Fungal endophytes are known to aid in herbivory defense (e.g., [25,26]), increase plant biomass and pathogen defense [27], and provide habitat-specific stress tolerance [28]. Fungal endophytes, along with arbuscular mycorrhizae and ectomycorrhiza, were previously identified from roots of the carnivorous plant *Drosera rotundifolia* (sundew) [29]. Similar to *D. rotundifolia*, fungal endophytes were found to colonize other carnivorous plants such as the pitcher plant *Nepenthes khasians* [30], the butterwort *Pinguicula moranensis* [31], and, more recently, leaves and traps of *D. muscipula* [32]. The role of many of these endophytes remains unclear. Recent studies, however, revealed that fungal endophytes found in leaf mucilage of *Drosera* spp. stimulate insect digestion [33], while root endophytes of *D. rotundifolia* were postulated to aid in stress response to the nutrient poor and acidic soils of *D. rotundifolia* native habitat [29]. It is likely based on these previous studies that native *D. muscipula* harbor both mycorrhizae and fungal endophytes in root structures to augment nutrient acquisition and/or aid in the stress response resulting from the nutrient poor acidic soils of *D. muscipula*’s native environment. In the present study, we present the first investigation of fungal associations of native *D. muscipula* roots in an effort to discern factors that may influence conservation and successful transplantation of native *D. muscipula* populations.

## 2. Materials and Methods

### 2.1. Collection of Roots and Soil

Roots of *D. muscipula* plants were sampled from two different populations within Lewis Ocean Bay Heritage Preserve (LOBHP), located in Horry County, South Carolina (collection permit: SC-77-2021). Exact location information of *D. muscipula* populations is not disclosed for protection of native *D. muscipula*. Plants sampled from Population 1 were collected from an area that had not experienced a prescribed burn within two years prior to collection. Plants sampled from Population 2 were collected from an area located on the edge of Carolina Bay near the water table level that had experienced a prescribed burn within two years prior to collection. Three plants of each population were sampled from within 30 cm of each other for a total of six plants sampled. Three roots were sampled for each plant. No plants were removed from the native environment. Only root segments were collected after excision with remaining root structure covered with soil to minimize harm to native *D. muscipula* populations. Each root sample was cut approximately three centimeters from the root tip of each plant. All root segments collected were placed on dry ice immediately following extraction from the native soil. During root extraction, two grams of soil surrounding each plant root system, approximately five centimeters in depth, were collected for identification of fungi in rhizosphere compared to mycorrhizae and fungal endophytes directly hosted in *D. muscipula* roots tissue (*n* = 6 soil samples). Soil was extracted by shovel, weighed on a scale, and then placed in a 15 mL tube. Root samples from each collection were stored at −80 °C for permeant storage and soil samples were stored at 4 °C. Soil collected from a site at the entrance of LOBHP where *D. muscipula* were not present in the environment and the plant community composition differed from the plant community found within *D. muscipula* populations sampled in the current study was collected as an environmental control (EC) for rhizosphere fungal analysis. The environmental control soil sample was collected in the same manner as the other soil samples, 2 grams of soil collected at approximately 5 centimeters of depth, soil weighed, placed in a 15 mL tube, and then stored at 4 °C until utilized in experimentation.

### 2.2. Microscopy

Trypan blue staining methods were used to visualize arbuscular mycorrhizal fungi (AMF) formations within *D. muscipula* root tissue. AMFs belong to the phylum Glomeromycota and comprise the most common form of mycorrhizal fungal associations within land plant roots [34]. Thus, AMFs are the most likely candidates for mycorrhizal associations within *D. muscipula* roots. One three-centimeter root segment sampled from each plant collected was labeled segment one and then cut into three pieces and stained following a methodology from Moukarzel et al. [35] with extended time modifications. A 0.05% trypan blue lactoglycerol stain was selected for the optimal visual representation of possible fungal structures, including those of AMFs [35]. Trypan blue stain is known to be selective for fungal cell walls instead of plant cells [35]; therefore, fungal structures of interest could be confirmed by observation. In brief, root tissue samples were treated with approximately two mL of 70% ethanol per root piece and left at room temperature for 24 h. Ethanol was removed prior to clearing. Root samples were left in 10% KOH at 97 °C for one hour to initiate clearing of tannins precluding fungal identification [36]. KOH solution was decanted and root tissues treated with 3% H_2_O_2_ for one hour at room temperature. After the hydrogen peroxide treatment, root tissues were rinsed three times with dH_2_O. Root tissues were acidified by immersing root tissue in 1% HCl for 30 min with subsequent decanting of HCl. Following acidification, root tissues were stained with 0.05% trypan blue lactoglycerol (0.05% *w*/*v* dissolved in a solution of 5% lactic acid, 50% glycerol, and 45% sterilized Milli-Q dH_2_O) overnight at room temperature. After 24 h, trypan blue stain was decanted, and root tissues were submerged in a de-staining solution (1:1:1 lactic acid: glycerol: dH_2_O) for 24 h. After 24 h of de-staining, root tissues were stored at 4 °C until placed onto a microscope slide for further observation.

All samples were viewed via an Olympus BX51 microscope (Olympus, Waltham, MA, USA) using 400× and 600× magnification paired with an Olympus DP74 camera (Olympus, Waltham, MA, USA) for photography. Root tissue samples were placed onto microscope slides with one drop of the 1:1:1 lactic acid: lactoglycerol: dH_2_O solution to preserve the root tissue. Stained samples were analyzed for AMF structures such as vesicles, arbuscular formations, and aseptate hyphae. All three smaller pieces of each root segment were visualized to ensure replication of AMF assessment with the exception of plant A from Population 2. Root tissue loss from plant A of Population 2 did not allow for more than one piece of root tissue to be stained due to lack of available sample.

Root tissue collected from *A. thaliana* ecotype Columbia-0 (Lehle seeds, Round Rock, TX, USA) grown on MS salts + 1% sucrose agar served as the negative control for discernment of AMF structure via microscopy. *A. thaliana* has lost symbiosis-specific genes over evolutionary time supporting the inability for this plant species to form stable AMF associations [37]. Root tissue was harvested from adult *Solanum lycopersicum* plants grown in potting soil mix as the positive control for AMF formations. *S. lycopersicum* is a known AMF host plant species [38]. Root segments from *A. thaliana* and *S. lycopersicum* were harvested in the same manner as root tissue from *D. muscipula*; a single root for each plant was cut approximately 3 cm from root tip, divided into three one-centimeter segments for replication of AMF analysis by microscopy. Root sections were cleared and stained with trypan blue following [35] with extended time modifications as described previously.

### 2.3. Fungal Cultures

A second root segment from each plant sampled was rinsed with dH_2_O to remove residual soil then sterilized using 70% ethanol. Each root segment was then cut in half, where one section was placed directly on a potato dextrose agar (PDA) plate (the uncut section). The section that remained was cut in half laterally to split open the root and expose internal root tissue (the ‘cut’ section). Each half of the split root section was then plated approximately two centimeters apart from each other, directly onto a PDA plate. Fungal growth from the ‘uncut’ and ‘cut’ root sections were compared to discern surface fungal growth (ectomycorrhiza or other) versus fungal endophytic growth. Root sections plated on PDA petri dishes were left in a partially lit room, with the sample facing upward at 25 °C until fungal growth was visible. Each fungal culture on PDA petri dishes was photographed four, seven, and nine days after plating. Overgrown fungal cultures were re-streaked onto fresh PDA media and photographed again after additional four days. Furthermore, cultures were continuously re-streaked until isolation to a single morphologically pure culture (single morphotype) was established per PDA plate.

### 2.4. Extraction and Amplification of Fungal DNA for Species Identification of Fungal Endophytes

DNA was extracted from each isolated fungal morphology using approximately one milligram of fungal culture in the DNeasy^®^ UltraClean^®^ Microbial kit (Qiagen, Hilden, Germany). All steps for DNA extraction followed the manufacturer’s instructions with the following two modifications: (1) samples were incubated at 97 °C for ten minutes after addition of Solution SL to the PowerBead tube; (2) samples were air-dried for five minutes prior to final elution from the MB spin column to increase concentration. DNA quantity and quality were assayed using the NanoDrop^TM^ One^C^ Microvolume UV-Vis Spectrophotometer (Thermo Fisher^TM^, Florence, SC, USA) with quantity re-assessed for accuracy using the Qubit 2.0 Fluorometer with Qubit^TM^ dsDNA High Sensitivity Assay Kit (Invitrogen^TM^, Waltham, MA, USA). ITS3_KY02 (5′-GATGAAGAACGYAGYRAA-3′) and ITS4_KY03 (5′-CTBTTVCCKCTTCACTCG-3′) primers which target the Internal Transcribed Spacer region 2 (*ITS2*) fungal DNA barcoding region [39] or *Glomeromycota*-specific AML1 (5′-ATC AAC TTT CGA TGG TAG GAT AGA-3′) and AML2 (5′-GAA CCC AAA CAC TTT GGT TTC C-3′) [40] primers were used for the amplification of fungal DNA with products resolved by gel electrophoresis.

The ITS3_KY02 and ITS4_KY03 primers were previously shown to cover over 96% of all fungal genomes (under one-mismatch conditions) with the exception of non-Dikarya fungi which had a lower coverage of 77% for the reverse ITS4_KYO3 primer, while selectively not amplifying plant genomes [39]. The AML1 and AML2 primers were utilized to ensure coverage of non-Dikarya fungi such as *Glomeromycota* [40].

PCR reaction mix included 1× Phusion High-Fidelity PCR Master Mix with HF Buffer, 1 μM of each primer, and 2 ng of DNA template in the final reaction. Cycling conditions were the same for both primer sets and included an initial denaturing of 98 °C for 2 min, followed by 35 cycles of 98 °C 10 s, 47 °C 30 s, 72 °C 30 s, and a final extension at 72 °C for 10 min. PCR products were resolved by gel electrophoresis, cleaned from residual PCR components using the GenElute^TM^ PCR Clean Up Kit (Sigma-Aldrich, St. Louis, MO, USA) for ITS PCR products or 30% PEG 8000/30 mM MgCl_2_ Polyethylene Glycol (PEG) solution for AML PCR products, and sent to Eurofins Genomics (Louisville, KY, USA) for Sanger sequencing. PEG-clean-up of PCR products was performed using 4× volume of TE [10 mM Tris-HCl (pH 8), 1 mM EDTA] buffer followed by addition of 1/2 × volume of 30% PEG 8000/30 mM MgCl_2_. Components were centrifuged at maximum speed for 15 min, supernatant removed, and DNA pellet rehydrated in 20 μL of TE pH 8.0 buffer. Sequenced products were analyzed using the BLAST+ version 2.17.0 algorithm [41] to discern fungal species identity based on the closest sequence match in GenBank.

### 2.5. Rhizosphere Soil Samples

All soil samples were processed in duplicate for technical replication. An environment control sample collected from a site lacking *D. muscipula* served as a negative control for metagenomics analyses to discern ubiquitous fungi from LOHBP versus those in specific association with *D. muscipula* roots and the plant community surrounding *D. muscipula*. Environmental DNA was extracted from 250 milligrams of soil using the DNeasy PowerSoil Pro kit (Qiagen, Hilden, Germany) following the manufacturer’s instructions. DNA quantity and quality were assayed using the NanoDrop^TM^ One^C^ Microvolume UV-Vis Spectrophotometer (Thermo Fisher^TM^, Florence, SC, USA) with quantity re-assessed for accuracy using the Qubit 2.0 Fluorometer with Qubit^TM^ dsDNA High Sensitivity Assay Kit (Invitrogen^TM^, Waltham, MA, USA). Environmental DNA samples were sent to GENEWIZ^®^ from Azenta Life Sciences (South Plainfield, NJ, USA) for ITS-EZ fungal metagenomics identification. Azenta’s ITS-EZ service utilizes the ITS2 genomic region for amplification and identification of fungal species using the Illumina Miseq platform (Illumina, Santiago, CA, USA). Amplicons were analyzed by Azenta using the QIIME (1.9.1) platform for statistical analysis and UCHIME ‘Gold’ to filter for usable sequence data and sequence alignment. VSEARCH (1.9.6) was used for Operational Taxonomic Units (OTUs) discernment using 97% sequence identity in the UNITES ITS database (version 10.0; http://unite.ut.ee, 21 February 2024). Only unique sequences with a read > 1 were kept while all other sequences (read = 1 or chimeric sequences) were removed from the analysis. One representative sequence for each discerned OTU was processed through QIIME (1.9.1) for species identification. Additional taxonomic identification was performed by Azenta using the Bayesian algorithm in RDP classifier (2.2) using a 97% similarity threshold in the Greengenes database (http://qiime.org/home_static/dataFiles.html, 21 February 2024)/18S rRNA database/ITS database.

## 3. Results

### 3.1. Microscopy of Trypan Blue Stained D. muscipula Root Tissues

Arbuscules are the characteristic morphological formation by which AMFs are named. These tree-like formations are generated after cellular penetration of AMF in plant cells to increase surface area for resource exchange [42]. In addition to arbuscules, AMF may form storage vesicles, spores, and hyphal coils [42], all of which can be visualized by microscopy after trypan blue staining of plant root tissue [35]. AMF also can be distinguished from dark septate endophytes after trypan blue staining due to the aseptate nature of AMF hyphae [35,43].

**Figure 1:**
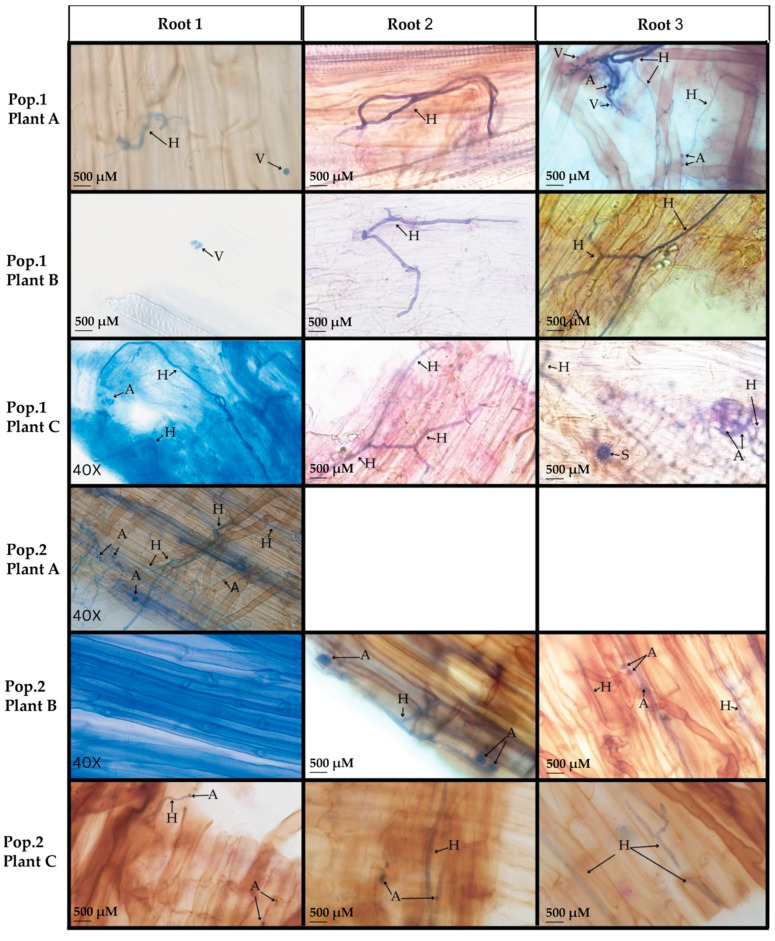
Trypan blue staining of *D. muscipula* roots for presence of AMF structures. One root segment from each plant was cut into three pieces (Root 1, 2, or 3) with each piece stained and analyzed separately for technical replication of the experiment. Pop. 1: roots of *D. muscipula* plants from Population 1 with individual plants labeled as A, B, and C; Pop. 2: roots of *D. muscipula* plants from Population 2 with individual plants labeled as A, B, and C. AMF structures noted as A for arbuscules, H for hyphae, S for spores, and V for vesicles. All images were taken at 60× magnification unless noted with 40× in the lower left of the panel. Scale bars are included for all 60× images.

**Figure 2:**
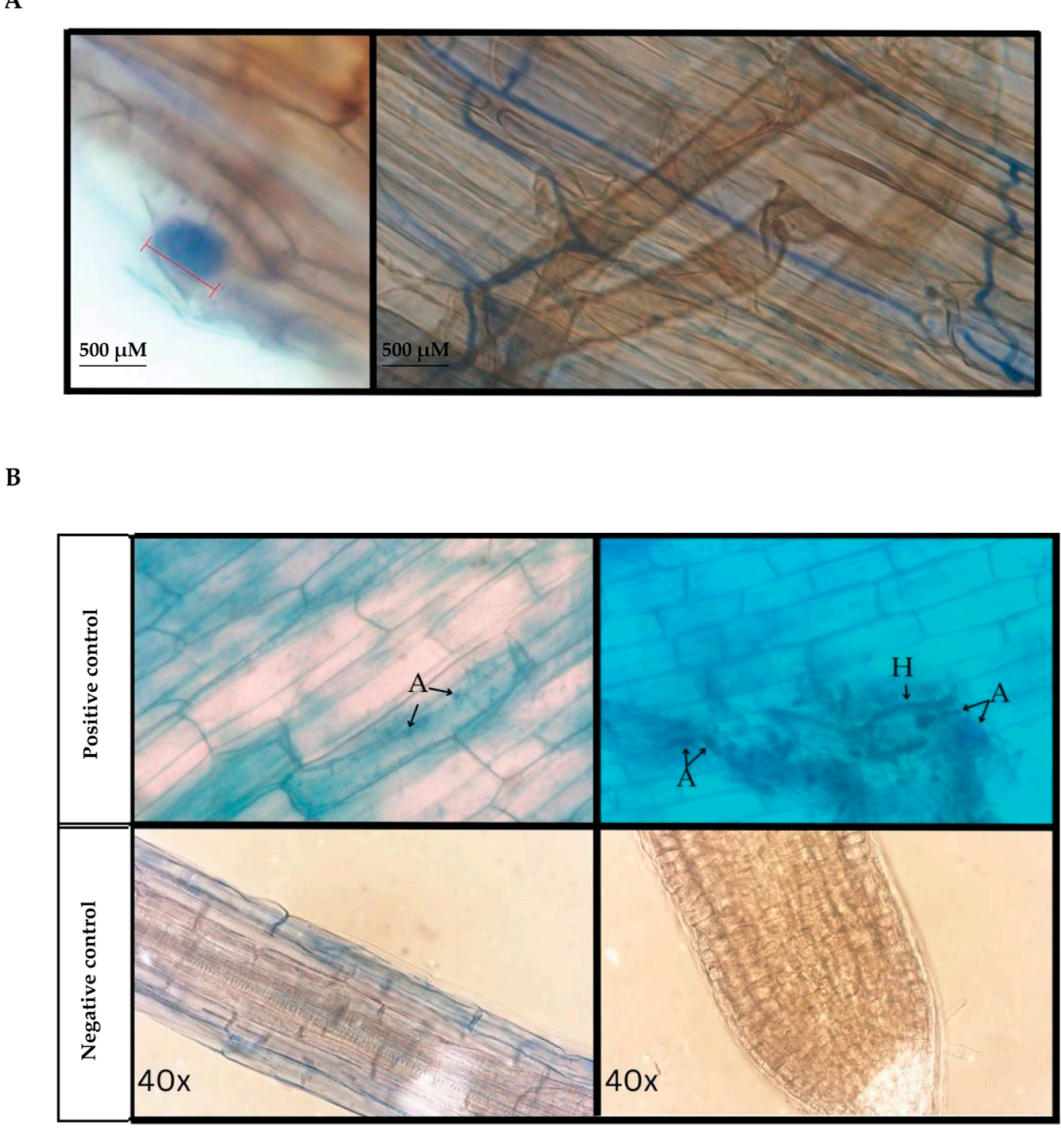
Trypan blue staining of *D. muscipula*, *S. lycopersicum*, and *A. thaliana* roots for presence of AMF structures. (**A**) Magnified image of an AMF arbuscule (left) and hyphae (right) from *D. muscipula* roots. (**B**) Trypan blue staining of positive (*S. lycopersicum*) and negative (*A. thaliana*) control root tissues used to discern AMF staining versus artifacts. AMF structures noted as A for arbuscules or H for hyphae.

A total of 16 *D. muscipula* root pieces (*n* = 6 plants with one root segment of each plant cut into three smaller pieces) were analyzed after trypan blue staining for evidence of AMF structures. Only one root piece was analyzed from Population 2 Plant A due to tissue loss. Out of these 16 root pieces, 15 root pieces (93.75% of root tissues analyzed) displayed intraradical structures such as arbuscules/coils, vesicles, and/or aseptate hyphae indicative of arbuscular mycorrhizae (Figure 1 and Figure 2A). No AMF structures were evident in one root piece from Population 2 Plant B (Figure 1 Root1). The other two pieces from this same root segment for Population 2 Plant B were observed to contain AMF structures (Figure 1 Roots 2 and 3). In total, AMF structures were evident after trypan blue staining of root tissue from all *D. muscipula* plants surveyed from both populations. All microscopy samples were compared to positive control (*S. lycopersicum*) and negative control (*A. thaliana*). Arbuscular formations were observed from root pieces of *S. lycopersicum*, a known AMF host [38,44], but not observed in root pieces of *A. thaliana,* a non-AMF host [37], (Figure 2B) supporting AMF structures evident from *D. muscipula* roots were not the result of artifacts or incorrect staining procedures.

### 3.2. Fungal Endophytes from D. muscipula Roots

**Figure 3:**
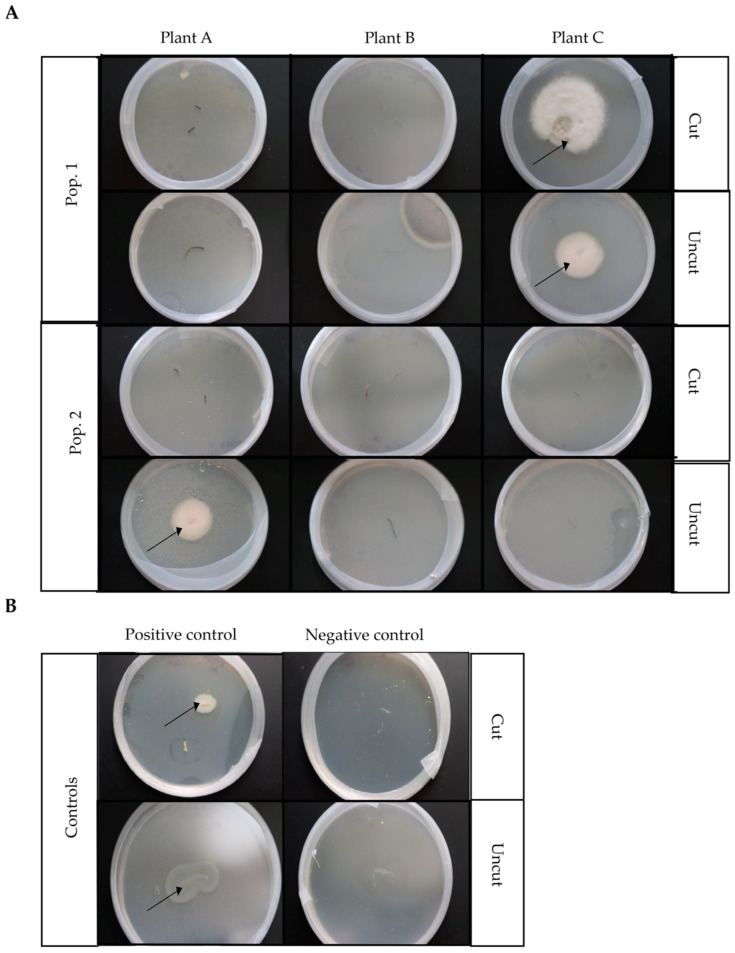
PDA-cultured endophytes from *D. muscipula* and control root sections. An approximately three-centimeter segment of root tissue from each of six *D. muscipula* plants (three plants per population) or controls (*S. lycopersicum* and *A. thaliana*) was cut in half with one half sterilized and placed directly on a PDA plate (the ‘uncut’ root piece) to culture potential surface fungi. The other half of the same root segment was sterilized and then cut laterally (the ‘cut’ root piece) to expose internal root tissue for endophytic fungal growth with each split half placed ~2 cm apart from each other on a PDA plate. (**A**) Results of fungal endophytic growth from *D. muscipula* root sections (cut or uncut) from each of three *D. muscipula* plants of each population. Pop1: fungal endophytic growth resulting from Population 1 Plants A-C, respectively, cut or uncut root sections, respectively. Pop2: fungal growth resulting from Population 2 Plants A–C, respectively, cut and uncut root sections, respectively. (**B**) Fungal growth after seven days incubation on PDA plates for positive (*S. lycopersicum*) and negative (*A. thaliana*) control cut and uncut root segments, respectively. Arrows indicate positive growth.

Root segments from each of the six *D. muscipula* plants sampled were plated on PDA media with one half of each root segment plated directly on PDA plates (noted as ‘uncut’) while the other half was cut laterally to expose interior root tissue (noted as the ‘cut’ root section). Both the ‘cut’ and ‘uncut’ root pieces were placed on PDA media to assess fungal growth. After seven days, fungal growth was evident from root sections of two out of the six *D. muscipula* plants sampled (Figure 3A). Fungal growth was evident from both the uncut and cut root pieces of Population 1 Plant C and from the uncut root piece of Population 2 Plant A (Figure 3A). Fungal growth on the PDA plate for Plant B Uncut (Figure 3A) did not originate from the root piece placed on this plate and, therefore, was considered a contaminant. Negative controls (root segments from *A. thaliana*) did not show fungal growth from either cut or uncut root pieces, confirming that fungal growth on plates stemming from root pieces was not due to general contaminants of the media or lab environment (Figure 3B). Only fungal growth originating from root pieces was considered positive for fungal endophytic presence and re-streaked for isolation of morphologically pure cultures. Positive fungal growth was evident from *S. lycopersicum* positive controls (Figure 3B).

**Figure 4:**
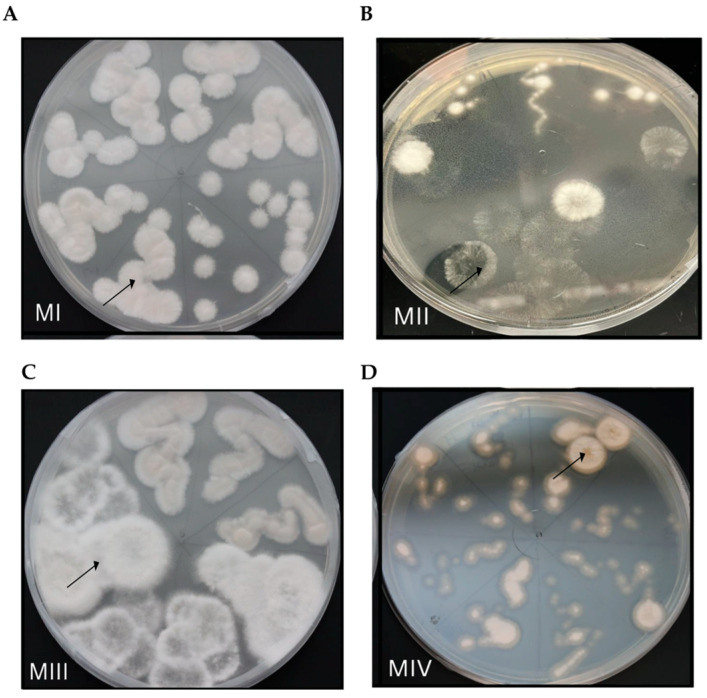
Isolated fungal morphologies from *D. muscipula* roots. Four distinct fungal morphologies were identified from *D. muscipula* roots after re-streaking for isolation. (**A**) Morphology I (MI) isolated from Population 1 Plant C cut and uncut root sections. (**B**) Fungal Morphology II (MII) and (**C**) morphology III (MIII), respectively, isolated from Population 1 Plant C cut root section. (**D**) Morphology IV (MIV) isolated from Population 2 Plant A uncut root section. Arrows indicate characterized morphologies.

Fungal growths evident from root segments (cut and uncut) from *D. muscipula* Population 1 Plant C and from the uncut root piece of Population 2 Plant A were further streaked onto new PDA plates until reaching a single morphotype for each fungal specimen per plate. Re-streaking efforts resulted in the identification of four distinct morphotypes (Figure 4). Three of these morphotypes originated from the same ‘cut’ root piece of Population 1 Plant C (Figure 4A–C, Table 1), while re-streaking of fungal growth on PDA plates from root pieces of Population 1 Plant C uncut root and Population 2 Plant A uncut root resulted in isolation of a single morphotype for each respectively (Table 1). Morphotype I (MI) distinguished by a solid white, raised, and fuzzy appearance (Figure 4A, Table 1) was identified from Population I Plant C cut and uncut root pieces. Fungal morphotypes MII and MIII in addition to MI were identified as outward fungal growth from Population I Plant C ‘cut’ root (Figure 4A–C). Morphotype IV, distinguished by a yellow color with translucent outer rim, was the only morphotype identified after 10 days of incubation and subsequent re-streaking efforts for isolation from Population 2 Plant A uncut root (Figure 4D, Table 1).

Primers targeted to the fungal ITS region of the nuclear genome (ITS3_KY02 and ITS4_KY03; [39]) and primers targeted to the 18s small subunit rRNA gene region of arbuscular mycorrhizal fungi (AML1 and AML2; [40]) were utilized in PCR with subsequent sequencing to identify fungal species. Morphotypes I, II, and III produced a PCR product of ~400 bp from amplification utilizing the ITS primers (Figure S1). Morphotype IV resulted in poor amplification with only a faint band produced with the ITS primers (Figure S1). A strong product of ~800 bp was produced for morphotype IV when amplified with AML1 and AML2 primers (Figure S2). DNA from fungal growth observed from PDA plates with roots from the *S. lycopersicum* positive control was also isolated and amplified using the ITS and AML primers. PCR product was only produced for positive control using the ITS primer set which produced multiple bands (Figure S1). PCR products from the positive control were not sequenced for fungal identity. Morphotype I isolated from cut and uncut root sections of Population 1 Plant C was determined to be *Penicillium rolfsii* after BLAST+ version 2.17.0 algorithm [41] sequence analysis through GenBank with 99.4% sequence identity to the query sequence. Morphotypes II and III isolated from cut roots of Population 1 Plant C were determined to be *Neopestalotiopsis* sp. based on BLAST+ version 2.17.0 algorithm [41] sequence analysis with a matching percent identity ranging from 96 to 99.7% depending on replicate PCR product sequenced (Table 2). Sequence analysis using the BLAST+ version 2.17.0 algorithm [41] in GenBank resulted in a 97.3% match for morphotype IV, the only fungal isolate identified from Population 2 Plant A, to *Penicillium limosum* (Table 2).

### 3.3. Fungal Rhizosphere Surrounding Native Venus’ Flytrap Roots of South Carolina

**Figure 5:**
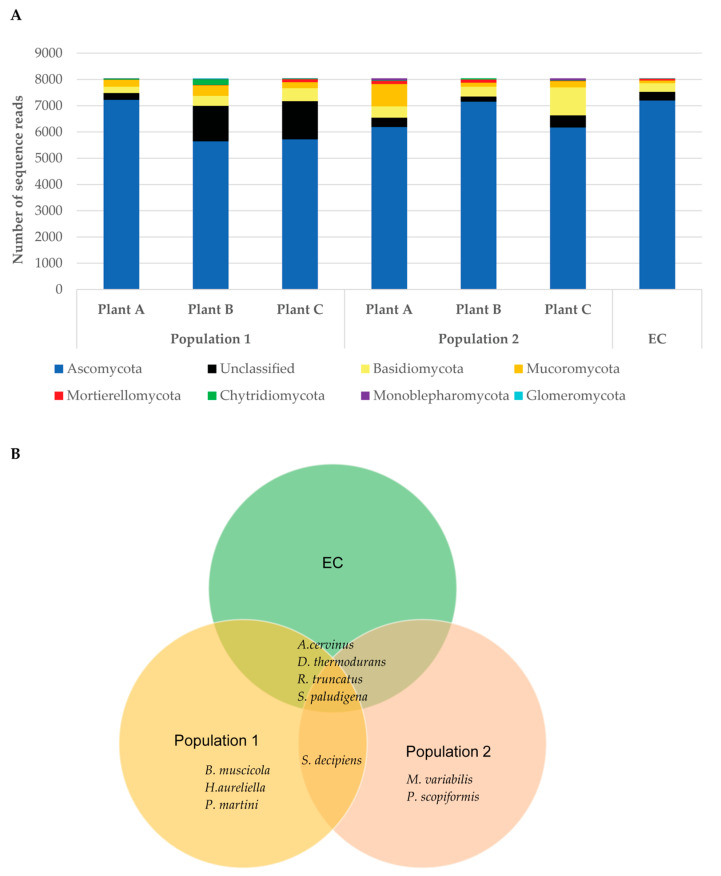
Fungal biome surrounding native *D. muscipula* roots as determined by metagenomics. (**A**) Relative abundance of identified soil fungi surrounding each *D. muscipula* plant categorized to phylum. Each sample was run in duplicate. Read number is the average read count for each phylum of both duplicates sequenced for each sample. (**B**) Venn diagram representation of rhizosphere fungal species of interest based on either increased presence only around *D. muscipula* compared to the environmental control or only found near *D. muscipula* roots in one of the two population study areas.

Metagenomic profiling of the soil fungal biome surrounding native *D. muscipula* roots resulted in the identification of 67 distinct species and a large category of unclassified species (Table S1). Lack of fungal classification for several species was due to previously uncharacterized or poorly annotated fungal genomes, or due to poor sequence resolution. Of these 67 species, 22 species only had sequence reads from the environmental control and were not indicative of fungal biome near either of the *D. muscipula* populations sampled in the current study (Table S1). Many species were in low abundance in the soils as indicated by low sequence reads (less than 10) and lack of presence in duplicate samples (Table S1). Three independent OTUs were described for *Acidothrix acidophila. Rhizopogon truncates*, *Devriesia thermodurans*, and *Ustanciosporium gigantosporum*, while two independent OTUs were assigned each for *Aspergillus cervinus*, *Diplogelasinospora grovesii*, and *Saitozyma podzolica* presumably due to either strain differences or sequencing limitations/errors. Data for all OTUs assigned to the same species were combined for the final data table under the assumption that these additional OTU designations were due to different strains of the same fungal species. The vast majority of discernable fungi species were in phylum Ascomycota but Chytridiomycota, Zygomycota, and Basidiomycota were represented to a lesser extent depending on sample (Figure 5A, Table S2). Of the 67 identified fungal species, ten species were of specific interest either due to increased presence only around *D. muscipula* compared to the environmental control or only found near *D. muscipula* roots in one of the two population study areas. These species included *Aspergillus cervinus*, *Blastobotrys muscicola*, *Devriesia thermodurans*, *Hyaloscypha aureliella*, *Meliniomyces variabilis*, *Penicillifer martinii*, *Phialocephala scopiformis*, *Sugiyamaella paludigena*, *Suillus decipiens*, and *Rhizopogon truncatus* (Figure 5B, Table S1). Although other fungi known to form plant-associated interactions such as the fungal endophyte *Penicillium melinii* [45] were identified from the rhizosphere surrounding *D. muscipula* roots, these other fungi were not found consistently in the same population and often with a very low number of sequence reads (Table S1) suggesting poor representation in the soil fungal biome. None of the 67 identified fungal species from the rhizosphere surrounding *D. muscipula* roots/ environmental control were the same as those identified from PDA fungal cultures (*Penicillium rolfsii*, *Neopestalotiopsis* sp, and *Penicillium limosum*, Table 2).

## 4. Discussion

### 4.1. Native D. muscipula Roots Harbor Arbuscular Mycorrhizae

Although the current study was limited to only six plants from a total of two populations, combined analyses of microscopy, plate cultures, and metagenomics from the current study provide strong support for the presence of fungal associations, both mycorrhizae and fungal endophytes, within native *D. muscipula* root tissues. Microscopy analysis of *D. muscipula* tissues resulted in identification of AMF-associated structures in 93.75% of root tissues (Figure 1). This discovery challenges the historical assumption that mycorrhizal associations are absent in Venus’ flytraps due to their evolved leaf traps used for prey capture [22,46]. Instead, it suggests that *D. muscipula*, like many other plants, evolved to establish a mutualistic relationship with fungi to possibly enhance nutrient uptake or combat stressful environmental conditions, such as the peat bog, nutrient poor soils where native Venus’ flytraps thrive.

Santiago et al. [24] previously identified mycorrhizal associations in the roots of a variety of greenhouse grown carnivorous plants including *Nepenthes sanguinea*, *Pinguicula laueana*, *Sarracenia purpurea*, *Darlingtonia californica,* and *Dionaea muscipula* [24]. Only a few studies, however, have surveyed and/or identified mycorrhizae in root or related structures of carnivorous plants in their native environment. Mycorrhizae were identified from corms of three native sundews species, *Drosera peltate* from northeast India [47], native *Drosera indica*, and *Drosera burmanii* of south India [48], as well as from the roots of native pitcher plants (*Nepenthes* spp.) from Indonesia [49]. Although Santiago et al. [24] identified mycorrhizae previously from greenhouse grown *D. muscipula*, greenhouse fungal associations may be impacted by other influences such as AMF host species in proximity that would not be found in the native environment, or AMF contaminated soils or other potting materials from the greenhouse. Our study presents the first data of mycorrhizae in native *D. muscipula* roots devoid of artificial influences providing concrete evidence that *D. muscipula* form these fungal associations as part of their natural physiology.

### 4.2. Fungal Endophytes of Native Venus’ Flytrap Roots

Three fungal endophytes were identified from PDA culture plates that included root segments from native *D. muscipula.* Two of the identified fungal endophytes were from the genus *Penicillium*. *Penicillium* is a large fungal genus comprised of both pathogenetic (e.g., [50,51]) and endophytic (e.g., [52,53]) species. *P. rolfsii* identified from root segments of Plant C from Population 1 was previously determined to be a fungal endophyte of papaya plants and contribute to pathogen defense in pitaya fruit [52]. The closely related species *P. limosum* was identified from root segments of Plant A from Population 2. Although these plants were not located in the same area or constitute the same population, both contained related *Penicillium* species. Both *P. rolfsii* and *P. limosum* are part of section *Lanata-divaricata* within the *Penicillium* genus [54]. Interestingly, members of the *Lanata-divaricata* section tend to be found in acidic soils such as those of *D. muscipula*’s native habitat [55] and most likely serve a role in acid tolerance in these soils.

*Neopestalotiopsis* sp. are both fungal endophytes and fungal pathogens of plants. *Neopestalotiopsis* sp. in *Cinnamomum loureiroi* leaves were determined to generate antimicrobial compounds [56] most likely for defense against bacterial plant pathogens. Conversely, *Neopestalotiopsis* spp. is a known fungal pathogen of strawberries (*Fragaria*) with infection resulting in stunted growth and rapid death of the plant within a month with severe infection (reviewed in [57]). Although we did not test to discern the role of *Neopestalotiopsis* sp. in *D. muscipula* roots, all plants examined in the current study appeared healthy with no observable phenotypic symptoms of fungal infection. Therefore, we propose that the *Neopestalotiopsis* sp. identified from fungal growth on PDA plates with native *D. muscipula* roots in our study served as a fungal endophyte of *D. muscipula* roots, not a pathogen. It is important to note, however, that in the present study we only confirmed the presence of these three fungal species in roots of *D. muscipula* plants. The functional role of *P. rolfsii, P. limosum,* and *Neopestalotiopsis* sp. in *D. muscipula* roots requires further investigation.

### 4.3. Soil Rhizome Surrounding Native D. muscipula Roots in South Carolina

The ecosystem surrounding native *D. muscipula* has been well described in previous literature [1,2]. To our knowledge, however, we are the first to describe the fungal biome surrounding native *D. muscipula* roots. Many interesting fungal species were identified from metagenomics analysis of rhizosphere soil samples that may indicate habitat- or plant-specific fungal communities. The fungus *S. decipiens* was identified in soil samples surrounding *D. muscipula* roots from both populations but not in the environment control (Figure 5B, Table S1). Habitat of *D. muscipula* Population 1 and Population 2 sampled in our study differed in hydrology and time since last prescribed burned but were otherwise characteristic of *D. muscipula* habitat. Both populations were located on the rims of Carolina Bays in acidic soils, intermediate to wetland bog and dry sandy terrain characterized by various pine species including *Pinus palustris* (longleaf pine) and *P. taeda* (loblolly pine) [1,2]. Population 2 was closer to the water table and had higher coverage of *Sphagnum* moss compared to Population 1. Interestingly, *S. decipiens* is an ectomycorrhiza associated with loblolly pine and generates several terpenes useful for host (loblolly pine) defense against pathogens and spore germination for *S. decipiens* [58]. Thus, the identification of *S. dicipiens* from our metagenomics data was suggestive of pine species present near sampled Venus’ flytraps rather than fungi of specific association to the Venus’ flytraps themselves.

Other fungal species of interest identified from metagenomics data included *A. cervinus*, *B. muscicola*, *D. thermodurans*, *H. aureliella*, *M. variabilis*, *P. martinii*, *P. scopiformis*, *S. paludigena*, and *R. truncatus* (Table S1). *B. muscicola*, *H. aureliella*, and *P.martini* were identified in soils surrounding all three Venus’ flytrap plants in Population 1 but not from Population 2 or the environmental control, while *M. variabilis* and *P. scopiformis* were found in soils surrounding all three sampled Venus’ flytraps from Population 2 but not in soils from Population 1 or the environmental control (Table S1). *H. aureliella*, although not directly identified as a mycorrhiza, is closely related to *Hyaloscypha hepaticicola* and other *Hyaloscypha* species demonstrated to form ectomycorrhizal structures with members of Ericaceae [59]. *M. variabilis* is also a part of the same clade as *H. hepaticicola* [59]. *M. variabilis* was previously shown to associate as an ectomycorrhiza of *Picea abies* (Norway Spruce) from the Czech Republic [59]. *M. variabilis* was also found associated with *Rhododendron albiflorum * roots in Canada [59]. *M. variabilis*, although identified from much more northern and colder habitats in previous studies [59] than the rims of Carolina Bays in South Carolina as in our study, has a worldwide distribution (e.g., [59,60]). Further, *M. viriabilis* is a prevalent ericoid mycorrhiza of many Ericaceae species including *Vaccinium* (blueberry) [60] that is a common plant of the Carolina Bays ecosystem and often seen in proximity to Venus’ flytraps [2].

*B. muscicola*, *P. martinii*, and *P. scopiformis* were identified in soils surrounding all three Venus’ flytraps sampled from one of the two populations and therefore may signify specific habitat differences between the two populations. *B. muscicola* was only found in soils of Population 1 (Table S1) and is associated with moss but not described previously as an endophyte or mycorrhizae [61]. Although *P. martinii* was previously isolated from stems and roots of the orchid *Paphiopedilum druyi* [62] suggesting a plant-associated function, no direct assessment has been conducted to demonstrate endophyte versus pathogenic characteristics of this fungus. Contrary to *B. muscicola* and *P. martinii*, *P. scopiformis* is a known fungal endophyte of coniferous trees which produces the insecticide rugulosin [63,64]. The combination of *B. muscicola*, *H. aureliella*, and *P. martini* identified from soils around Venus’ flytrap roots of Population 1 suggest Venus’ flytraps in this population were in close proximity to moss, orchids or other monocots, and a member of the Ericaceae family (most likely *Vaccinium*) characteristic of the floral communities typically found in the rims of the Carolina Bays ecosystem [2]. The identification of *M. variabilis* and *P. scopiformis* from soils surrounding Venus’ flytraps in Population 2 most likely reflect the presence of *Vaccinium* and coniferous trees such as loblolly and longleaf pine. The differences in soil fungal biome of each *D. muscipula* population suggest differences in plant community structure between the two populations but does not appear to signify any specific differences in soil pH, macronutrient, or other selective characteristic of fungal growth. The proximity of these mycorrhizae to *D. musciupla* may influence the ability of *D. muscipula* to act as an ectomycorrhizal host. Many plants only form mycorrhizal associations when in proximity to fungal networks of host plant species [19]. Thus, native *D. muscipula* may not be natural hosts for these ectomycorrhizal species but may form ectomycorrhizal and other fungal associations due to surrounding plant communities. However, the fact that arbuscular mycorrhizae were evident from roots of both native *D. muscipula* and greenhouse grown *D. muscipula* plants [24] support that *D. muscipula* is a host species for mycorrhiza.

Of the four remaining species of interest identified from our metagenomics data, only *R. truncates* is a fungus known to associate in a symbiotic relationship with plants. *R. truncates* is a known ectomycorrhiza of *Pinus* (pine trees) [65]. Members of the *Rhizopogon* genus are known to increase pine growth and may aid forestation efforts [66,67].

The other three fungal species of interest identified from the metagenomics analysis of soil surrounding native *D. muscipula* plants from LOHBP (*A. cervinus*, *D. thermodurans*, and *S. paludigena*) are not known to function as fungal endophytes or mycorrhizae but as common saprophytes in the soil [68,69,70]. The increased abundance of these saprophytes near *D. muscipula* may be attributed to the increased decaying matter in the soils due to Venus’ flytrap carnivory.

In summary, several known fungal endophytes and ectomycorrhizal fungal species were identified from soils surrounding *D. muscipula* roots in their native environment. The influence these fungi have on *D. muscipula* growth, vigor, and survival still needs to be assessed. None of the species identified were specific to carnivorous plants based on current literature but all have the potential to form fungal associations with Venus’ flytraps and may impact success of Venus’ flytraps in their native environment. Further, none of the fungal species identified from metagenomics data of the rhizosphere surrounding *D. muscipula* roots were the same as fungal species identified from cultured *D. muscipula* root tissues grown on PDA. It is possible that fungal species identified from cultured root tissue were host specific to *D. muscipula* rather than the surrounding plant community limiting concentration in the rhizosphere and critical abundance for metagenomics analysis. Further, fungal endophytes identified from PDA plate cultures were the result of fungal growth after a total of 10 days, possibly biasing sampling to fast-growing fungi. It is highly possible that longer growth times would enable identification of additional fungal species that may coincide with metagenomics identified fungal species from the *D. muscipula* rhizosphere.

## 5. Conclusions

Conservation of native *D. muscipula* requires understanding of the unique physiology and ecology of this carnivorous and iconic plant species including fungal relationships that contribute to plant health and vigor. In the current study we present evidence for the presence of mycorrhizae and fungal endophytes in root structures from native *D. muscipula*. We further determined fungal species in the surrounding rhizosphere of *D. muscipula* roots that may impact growth, pathogen defense, and nutrient acquisition of this and the surrounding plant community. Such information is important for conservation and management of native species. Biofertilizers which incorporate microorganisms for increasing nutrient acquisition to plant root structure are becoming prominent in agricultural practice to increase vigor of crops (e.g., [71,72]). Such methods could also be applied in conservation when trying to augment new habitat for transplanted plant species or preserve rare and endangered species in botanic gardens. However, implementation of biofertilizers first requires knowledge of host species for mycorrhizae and fungal endophytes as well as the species of these fungi most beneficial for the host plant. Our work hopes to contribute to this knowledge to aid conservation of native Venus’ flytraps. Although we identified several potentially beneficial fungal species of *D. muscipula* roots, it is important to note that many fungi, including mycorrhizae, may be detrimental or beneficial depending on host or nonhost species (e.g., [19]). The specific influence of the identified fungal species from our study in *D. muscipula* physiology requires further study to discern impact, benefit, or detriment to *D. muscipula* survival. Further, a broader sampling of plants is suggested for future studies to assess if fungal endophytes identified in our study are prominent in other *D. muscipula* populations or limited to the few plants in our study. Longer incubation of fungi on PDA media is also recommended for possible discovery of additional slow-growing fungal endophytes that may impact *D. muscipula* root physiology.

## Figures and Tables

**Table 1:** Morphological analysis of fungal morphotypes isolated from roots, both cut and uncut, of *D. muscipula* plants placed on PDA media and given an initial culture period of 10 days followed by subsequent streaking for isolation of morphotypes.

Plant	Morphotype	Description
Pop. 1 Plant C cut root	I	Solid, white, fuzzy, circular formation with circles connecting in line, slightly raised
Pop. 1 Plant C cut root	II	Branching, transparent white fuzzy circular formation, with little to no center growth, very slightly raised, formed in connecting clusters
Pop. 1 Plant C cut root	III	Solid, white, fuzzy, uneven circular formation, raised
Pop. 1 Plant C uncut root	I	Solid, white, fuzzy, circular formation with circles connecting in line, slightly raised
Pop. 2 Plant A uncut root	IV	Solid, yellowish, fuzzy semicircular formation with translucent outer rim surround the circular formation, slight yellow indent center in circle formation

**Table 2:** Morphotype fungal identity discerned by sequence analysis using the BLAST+ version 2.17.0 algorithm [41] in GenBank.

Morphotype	Species	Phylum	% Identity ^1^	Region
MI	*Penicillium rolfsii*	Ascomycota	99.4	ITS
MII	*Neopestalotiopsis* sp.	Ascomycota	96–99.7	ITS
MIII	*Neopestalotiopsis* sp.	Ascomycota	99.4–99.7	ITS
MIV	*Penicillium limosum*	Ascomycota	97.3	18s rRNA

^1^ % Identity of input sequence to species as determined by BLAST+ version 2.17.0 algorithm [41] search in GenBank.

## Data Availability

The original contributions presented in this study are included in the article/Supplementary Material. Further inquiries can be directed to the corresponding author.

## Supplementary Materials

The following supporting information can be downloaded at https://www.mdpi.com/article/10.3390/microorganisms13102269/s1, Figure S1: Resolved ITS PCR products (~400 bp) from DNA isolated from each fungal morphotype; Figure S2: Resolved AML PCR products (~800 bp) from DNA isolated from each fungal morphotype; Table S1: Fungal species identified from rhizosphere surrounding *D. muscipula* roots; Table S2: Raw OTU data table from rhizosphere metabarcoding.

## Abbreviations

The following abbreviations are used in this manuscript:

LOBHP	Lewis Ocean Bay Heritage Preserve
EC	Environmental Control
Pop1	Population 1
Pop2	Population 2
Pl.	Plant (A, B, C of each population)
PDA	Potato Dextrose Agar
AMF	Arbuscular mycorrhizal fungi
PEG	Polyethylene Glycol
OTU	Operational Taxonomic Unit
