# Supplementary material for: It’s a Question at the ‘Root’ of the Problem: Fungal Associations of Dionaea muscipula (Venus’ Flytrap) Roots in Its Native Habitat
Source: Microorganisms. 2025 Sep 27;13(10):2269. doi: 10.3390/microorganisms13102269 (PMC12566226; doi:10.3390/microorganisms13102269)
Supplement: Supplementary file 1 [file microorganisms-13-02269-s001.zip › Figure S2 AML PCR.pdf]

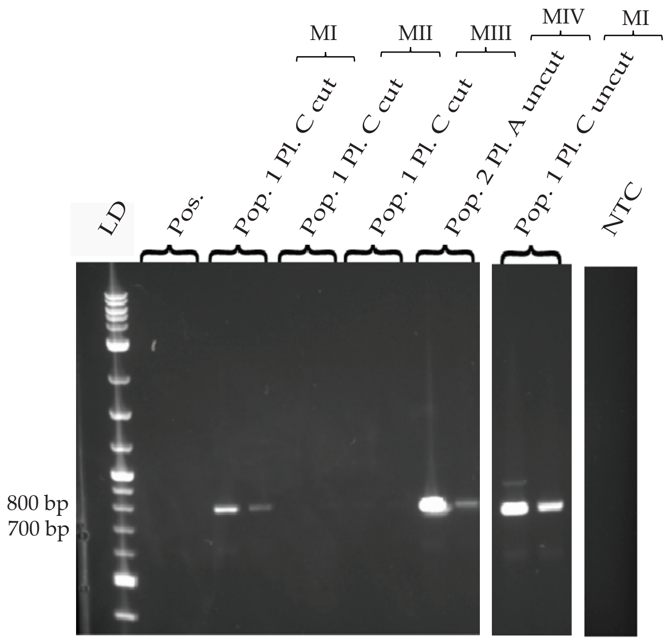

Figure S2. Resolved AML PCR products (~800 bp) from DNA isolated from each fungal morphotype. All fungal cultures were isolated to a single morphotype in duplicate to ensure the same morphotype would arise each time. Fungal DNA from each duplicate culture was isolated using the DNeasy® UltraClean® Microbial kit (Qiagen, Hilden, Germany) and used in PCR with the AML1 and AML2 [40] primer pair. PCR products from each duplicate were cleaned of residual PCR components and sent for sequencing with the expectation that both duplicates should represent the same fungal species/ isolate. LD: Tridye 1 Kb plus DNA ladder (NEB, Ipswich, MA, USA)., Pop. 1 Pl. C: Population 1 Plant C, Pop. 2 Pl. A: Population 2 Plant A. NTC: No template control of the PCR reaction. Origin of amplified product as from DNA isolated from streaked fungal morphotypes resulting from the 'cut' or 'uncut' root section of each plant root is noted in lane labels.
