# Supplementary material for: It’s a Question at the ‘Root’ of the Problem: Fungal Associations of Dionaea muscipula (Venus’ Flytrap) Roots in Its Native Habitat
Source: Microorganisms. 2025 Sep 27;13(10):2269. doi: 10.3390/microorganisms13102269 (PMC12566226; doi:10.3390/microorganisms13102269)
Supplement: Supplementary file 1 [file microorganisms-13-02269-s001.zip › Table S1.pdf]

Table S1. Fungal species identified from rhizosphere surrounding *D. muscipula* roots. Number of reads for each species is given. If found in both duplicates of the same sample, an average number of reads for both duplicates is shown. If only found in one of the two duplicates, the number of reads shown represents only the duplicate in which that species was evident.

Phylum	Species	Pop. 1A [a]	Pop. 1B [a]	Pop. 1C [a]	Pop. 2A [a]	Pop. 2B [a]	Pop. 2C [a]	Found in duplicate [b]	EC [c]
Ascomycota	*Acidomelania panicicola*	4	1	36	0	0	1	No	0
Ascomycota	*Acidothrix acidophila*	1	2	0	0	0	0	No	2
Ascomycota	*Acremonium persicinum*	3	0	1	0	0	0	No	0
Ascomycota	*Annulohypoxylon truncatum*	0	0	1	0	0	0	No	0
Ascomycota	*Aphanoascus cinnabarinus*	0	3	0	0	0	0	No	0
Ascomycota	*Aspergillus cervinus*	20.5	50.5	22.5	225	123.5	10	Yes	27
Ascomycota	*Blastobotrys muscicola*	1	3	7	0	0	0	Yes	0
Ascomycota	*Byssochlamys spectabilis*	0	0	5	0	0	0	No	0
Ascomycota	*Candida zeylanoides*	2	2	0	0	0	0	No	0
Ascomycota	*Conlarium dupliciascosporum*	0	0	0	0	0	0	No	2
Basidiomycota	*Craterellus tubaeformis*	0	0	1	0	156.5	706.5	Yes	1
Ascomycota	*Creosphaeria sassafras*	0	2	0	0	0	0	No	0
Ascomycota	*Dactylella zhongdianensis*	0	0	0	0	0	0	No	3
Ascomycota	*Devriesia thermodurans*	5.5	14.5	11.5	4	2	15.5	Yes	1
Ascomycota	*Diplogelasinospora grovesii*	3	0	12.5	0	0	0	Yes	0
Ascomycota	*Fusarium oxysporum*	0	0	0	0	0	0	No	1
Basidiomycota	*Gjaerumia minor*	0	0	0	0	1	4	No	0
Ascomycota	*Halokirschsteiniothelia maritima*	0	0	0	0	0	0	No	2
Ascomycota	*Hyaloscypha aureliella*	2	3	11	0	0	0	Yes	0

Ascomycota	*Hypomyces ochraceus*	0	0	0	0	0	0	No	7
Basidiomycota	*Jaapia ochroleuca*	0	0	0	0	0	1.5	Yes	0
Ascomycota	*Lecythophora fasciculata*	0	0	0	0	0	0	No	19
Basidiomycota	*Malassezia restricta*	0	0	0	0	0	0	No	1
Ascomycota	*Meliniomyces variabilis*	0	0	0	4.5	6.5	22	Yes	0
Ascomycota	*Monascus eremophilus*	0	0	0	0	0	0	No	1
Ascomycota	*Monocillium indicum*	0	0	3	0	0	0	No	3
Zygomycota, Mortierellomycota	*Mortierella gemmifera*	0	0	0	44.5	1	0	Yes	0
Zygomycota, Mortierellomycota	*Mortierella humilis*	0	0	2	0	0	0	Yes	0
Zygomycota, Mucoromycotina	*Mucor moelleri*	0	0	3	0	0	0	No	0
Ascomycota	*Myxocephala albida*	0	0	0	0	0	0	No	1
Ascomycota	*Neopestalotiopsis foedans*	0	1	0	0	0	0	No	2
Ascomycota	*Penicillifer martinii*	1	4.5	23.5	0	0	0	Yes	0
Ascomycota	*Penicillium lividum*	0	0	0	0	0	1	No	4
Ascomycota	*Penicillium melinii*	12	3	0	1	0	25.5	Yes	5
Ascomycota	*Penicillium spinulosum*	0	0	0	0	0	0	No	19
Ascomycota	*Penicillium sumatraense*	0	0	0	0	0	0	No	1
Ascomycota	*Phialocephala scopiformis*	0	0	0	7	1	60	Yes	0
Ascomycota	*Phialophora cyclaminis*	0	0	0	0	0	0	No	1
Basidiomycota	*Phragmotaenium fulvescens*	0	0	4	0	0	0	No	0
Basidiomycota	*Pisolithus arhizus*	0	0	0	2	0	0	No	1
Ascomycota	*Plenodomus biglobosus*	0	0	0	0	0	0	No	36
Ascomycota	*Purpureocillium lilacinum*	0	0	0	0	0	0	No	3
Ascomycota	*Rasamsonia brevistipitata*	0	0	0	1	0	0	No	1
Basidiomycota	*Rhizopogon evadens*	0	0	0	0	2	0	No	0
Basidiomycota	*Rhizopogon*	3	14	24	81.5	3	14.5	Yes	5

	truncatus								
Basidiomycota	*Rhodosporidiobolus nylandii*	0	0	0	1	0	0	No	0
Basidiomycota	*Saitozyma podzolica*	102.5	117	215.5	11.5	13	49	Yes	187
Ascomycota	*Solheimia costispora*	0	0	0	0	0	0	No	9
Basidiomycota	*Solicoccozyma keelungensis*	0	0	0	1	0	0	No	0
Basidiomycota	*Solicoccozyma terrea*	0	0	0	0	0	0	No	23
Basidiomycota	*Solicoccozyma terricola*	0	0	0	0	0	0	No	1
Chytridiomycota	*Spizellomyces acuminatus*	0	0	0	0	0	0	No	1
Basidiomycota	*Sporobolomyces johnsonii*	0	0	0	0	0	1	No	0
Ascomycota	*Sugiyamaella paludigena*	6.5	7.5	15	150	87.5	122.5	Yes	1
Basidiomycota	*Suillus cothurnatus*	0	0	2	0	0	0	No	0
Basidiomycota	*Suillus decipiens*	8	20	14	10.5	18.5	5.5	Yes	0
Ascomycota	*Talaromyces amestolkiae*	0	0	0	0	0	7.5	Yes	0
Ascomycota	*Talaromyces assiutensis*	0	3	6	1	0	0	No	2
Ascomycota	*Talaromyces austrocalifornicus*	0	6	0	0	0	0	No	0
Ascomycota	*Talaromyces purpureus*	0	0	0	0	0	0	No	22
Ascomycota	*Talaromyces subinflatus*	6.5	3.5	4	1	0	4	Yes	2
Basidiomycota	*Thelephora corticioides*	0	0	2	0	0	0	No	0
Zygomycota, Mucoromycotina	*Umbelopsis dimorpha*	0	0	0	0	0	0	No	1
Zygomycota, Mucoromycotina	*Umbelopsis ovata*	0	0	0	1.5	0	1.5	Yes	0
Basidiomycota	*Ustanciosporium gigantosporum*	2	0	0	3.5	0	1.5	Yes	1
Ascomycota	*Wilcoxina mikolae*	0	0	0	0	0	0	No	21
Ascomycota	*Zymoseptoria brevis*	1	0	0	0	0	0	0	0
	Unclassified	7872	7766	7649	7495.5	7630	6934.5	Yes	7622

[a] Specific plant from population where fungal species was identified from surrounding soil to the roots. Number is the population number (1 or 2). Specific plant is noted by a letter with n= 3 plants per a population (plants A, B, and C).

[b] Notation if species was found in both duplicates of soil samples processed for metagenomics species identification.

[C] Environmental Control (EC): Soil sample taken in an area that did not contain *D. muscipula* plants for comparison of fungal biome.
